# ‘Preserve or perish’: food preservation practices in the early modern kitchen

**DOI:** 10.1098/rsnr.2019.0004

**Published:** 2019-07-10

**Authors:** Lucy J. Havard

**Affiliations:** University College London, Science and Technology Department, 22 Gordon Square, London WC1H 0AH, UK

**Keywords:** early modern kitchen, recipe books, domestic experimentation, food preservation

## Abstract

Early modern manuscript recipe books have become increasingly popular sources for historical research over recent years. Extensive compilations of food recipes, medicinal remedies and household tips, these manuscripts provide rich, multi-faceted opportunities for historical study and discussion. This paper utilizes recipe books as a means to examine contemporary food preservation practices. Through detailed textual analysis of these manuscripts, and the reconstruction of early modern preserving recipes, I explore the explicit and tacit ‘domestic knowledge’ required for food preservation. I argue that, rather than being a straightforward activity, this was a complex process requiring significant judgement, intuition and experience on the part of the housewife. Preservation was an experimental practice that might be considered under the umbrella of early modern natural philosophy, and the housewife was a legitimate actor in the associated knowledge production.

*The inward and outward Vertues which ought to be in a compleate Woman.*^1^

Over recent years, the early modern kitchen has become recognized as an important area worthy of historical study.^2^ It was a space typically dominated by women, who, in comparison to men, wrote little that has survived to the present day.^3^ With the rise of feminist and gender history, what happened behind the kitchen doors has become an ever more pressing question, with historians seeking to discover the untold stories of ordinary women.^4^

Preserving food in the early modern period was very much a practical necessity. It was the early modern housewife who shouldered the main responsibility in ensuring adequate provision of food for the household.^5^ This is made clear in Gervase Markham's domestic manual *The English Huswife* (1615), which stipulates the virtues of a ‘compleate woman’. She must have:

skill in … banqueting-stuffe, ordering of great feasts, preserving of all sorts of wines …, distillations …, the knowledge of dayries, office of malting, oats, their excellent uses in a family, brewing, baking, and all other things belonging to an houshold.^6^

In order to satisfy these expectations, the housewife had to understand and utilize the appropriate methods and means of preservation in the home.

There is an extensive literature on the topic of preservation in multiple contexts; not only within the realms of food, but also in medicine, anatomy and natural philosophy. C. Anne Wilson, Peter Brears and Joan Thirsk provide detailed accounts of the motives, means and methods of food preservation in the early modern period.^7^ The practical need for preservation is reflected in the extensive range of foods preserved in published and manuscript recipe books: fruits such as cherries, apricots and quinces; nuts such as walnuts and almonds; meat that might be salted, dried or potted; flowers such as candied roses; and vegetables such as pickled cucumbers.^8^ Anne Stobart has detailed some of the methods used to preserve food, by employing sugar, alcohol or distillation.^9^ Sometimes a combination of methods were used to preserve: Thirsk describes the ‘seething’ of meat, ‘potting it in vinegar with juniper seeds and salt, and keeping it in a cool cellar’ to maintain freshness over the summer months.^10^ Early moderns certainly had to be inventive when preserving, and they relied on a wide variety of techniques, as the recipe books demonstrate.

Preservation in early modern natural philosophy has received ample attention over the past decade: for example, through exploration of the preservation of anatomical specimens for the purposes of anatomy instruction, or the preservation of animals through taxidermy in cabinets of natural curiosities.^11^ Daniel Margócsy highlights this crossover, describing how taxidermists used pepper and tobacco dust to preserve animal hides, while small animals were bottled up in jars of alcohol, much like the pickled cucumbers of recipe books.^12^

As these authors have shown, there was an early modern appreciation that preservation practices interacted with the production of natural knowledge, so that there was no distinct border between ‘scientific’, ‘medical’ and ‘domestic’ knowledges. At the same time, exactly what kinds of knowledge were entailed in domestic preservation is less clear. The aim of this essay is to develop a more precise picture of the ‘domestic knowledge’ required for early modern food preservation. I define ‘domestic knowledge’ as the tacit, intuitive knowledge that was needed to manage a household in the early modern period.

Recipe books offer intriguing evidence of ‘domestic knowledge’. They comprise compilations of food recipes, medicinal remedies and household tips and aptly reflect the wide variety of activities and tasks that took place in the early modern home. This essay will focus on early modern food preservation, approaching this concept from two angles: first, it recounts the reconstruction of recipes, in this case to preserve walnuts, in order to better appreciate the skills and ‘tacit’ knowledge required for preservation practices; and second, it offers a textual study of a series of recipe book manuscripts, in order to afford some context in understanding the methods and motives behind preservation.^13^

Historians of early modern recipes recognize that more knowledge was required to cook successfully than what was simply written on the page. This ‘unwritten knowledge’ can be better appreciated by making the recipes. Numerous scholars have adopted an ‘experimental approach’, and proponents of ‘reconstruction’ such as Otto Sibum have insisted that it is not equivalent to replication, because it is impossible to replicate an experiment in its original historical context. Rather, reconstruction is a heuristic, a means of raising questions through performance and action that textual analysis might overlook.^14^

Historians of food and medicine have revealed how recipes were a vital part of the knowledge economies of early modern households.^15^ Many have adopted a practical approach in studying recipes, remaking early modern dishes to learn about their techniques and products.^16^ In this essay, I use recipe reconstruction of ‘preserved walnuts’ to make apparent the different kinds of knowledge preservation required and particularly the aspects of that knowledge that were not recorded in recipes. These are what I will call ‘assumed knowledge’, ‘thrift-orientated knowledge’ and ‘experiential knowledge’. I argue that preservation was a complex activity that required skilled observation, judgement and an extensive knowledge base. Early modern cooking and preserving was an epistemological practice in its own right, and might even be seen as a prerequisite to the natural philosophical activities of men in male-dominated institutions such as the Royal Society.^17^ In this way, preservation practices can be granted a legitimate place under the umbrella of early modern natural philosophy and associated knowledge production.

This paper is based on a sample of recipe books from the Wellcome Library in London. The sample is limited (twelve recipe books), and only a fraction of the full collection, but it was felt to be representative of the recipes to preserve walnuts. Of course, the preserving knowledge required for walnut recipes may not be extendable to other foodstuffs: the techniques used to preserve fruits and vegetables were markedly different from those for meat and fish, for example. Nevertheless, the types of knowledge involved might usefully be generalized to other recipes.

The first section of this paper focuses on reconstruction as a means to identify the ‘tacit’ domestic knowledge needed for food preservation in the early modern kitchen. The second section provides some context by exploring the housewife's motivations behind recipe book compilation, and discussing in greater detail the primary source material and the concept of ‘domestic knowledge’. The third section discusses the objects of preservation and describes the domestic knowledge required for preserving, while the final section considers how preservation was ‘experimental’.

## Reconstructing the past

Food preservation involved much more than the recipes recorded in recipe books. Householders needed a variety of knowledges, skills and techniques to preserve foods. Textual analysis can therefore provide only a partial picture of the knowledge needed to undertake preservation, and for this reason alternative methods might serve to reveal the hidden and tacit dimensions of preserving techniques.

An ‘experimental approach’ was adopted in this project as a means to elucidate these hidden, tacit elements of preserving. Hjalmar Fors, Larry Principe and Otto Sibum furnish us with a comprehensive definition of an ‘experimental approach’, describing it as ‘one of many possible historical tools whose purpose is to aid us in our endeavour to understand the past’, offering ‘fresh and potentially vivid approaches to what historical actors were doing and thinking, as well as why’.^18^ Thijs Hagendijk is similarly of the opinion that such ‘experimental history’ can ‘significantly enhance more traditional humanistic methods such as close-reading and archival research’.^19^ As Hagendijk explains, there are a variety of different terms used to describe the associated varying methodological approaches, including ‘restaging’, ‘reconstruction’ and ‘re-enactment’.^20^ My chosen methodological approach is ‘reconstruction’ and this was strongly influenced by Columbia University's ‘Making and knowing project’, founded by the historian Pamela Smith in 2014. Smith describes ‘reconstruction’ as involving ‘both subjective action, self-reporting, and the manufacture of evidence by the historian in the present’.^21^ Although the ‘Making and knowing project’ largely focuses on technical recipes for metal casting and mould-making rather than recipes for food, Smith notes that the ‘reconstruction’ of these recipes from a sixteenth-century manuscript ‘appeared promising as a means to begin to answer some of the questions that recipe compilations present to the historian’.^22^

Editor Klaus Staubermann's volume *Reconstructions: recreating science and technology of the past* brings together a collection of material and immaterial reconstructions from the Iron Age to the nineteenth century by scholars from varying areas of expertise.^23^ These papers highlight the value of reconstructions in the history of science and technology and their ability to recover ‘lost’ knowledge and to answer questions as well as raise them.

Historical recipe reconstruction has enjoyed much attention on various social media platforms, and this helped to influence the inception of this project. Online blogs such as ‘The recipes project’, ‘Early modern recipes online collective’ (EMROC) and ‘Cooking in the archives’ also have a strong presence on Twitter, Instagram and Facebook, making their work infinitely accessible to the modern-day researcher.^24^

Preserved walnuts were chosen as the recipe to reconstruct for several reasons. First, there was an abundance of recipes for this delicacy in the Wellcome Library's recipe books, making them particularly accessible for this purpose. Joan Thirsk describes how walnut trees were ‘highest in fashion’ of all the nut trees in the early modern period; they were popular on account of their fruit and their wood. Huge amounts of walnuts were picked and they were eaten ‘fresh, preserved and candied’.^25^ In some cases, multiple recipes for preserved walnuts may be found in the same manuscript: Elizabeth Jacob's recipe book contains no fewer than twelve.^26^ This provides ample opportunity to examine both the similarities and differences between such recipes and the variations that exist within the bounds of a single manuscript. Recipe repetition in itself also encourages ‘trial and error testing’, as Ann Blair has shown, and this will be discussed in greater detail in the final section of this essay.^27^

The second reason for concentrating on walnuts is that early moderns considered them an important food to preserve because of their specific health benefits. As the French philosopher Michel Foucault noted in his discussion of Renaissance knowledge, the concept of ‘resemblance’ played a key role in understandings of nature, so that, for example, in the seventeenth-century ‘episteme’, walnuts were particularly effective in treating ailments of the head, owing to their resemblance to the human brain.^28^ The resemblance between walnuts and the brain was a ‘signature’, implanted by God, teaching people how to use them to their benefit.^29^ While this use of walnuts is not mentioned explicitly in the manuscripts, the frequency of recipes is suggestive of their value.

**Figure 1.**
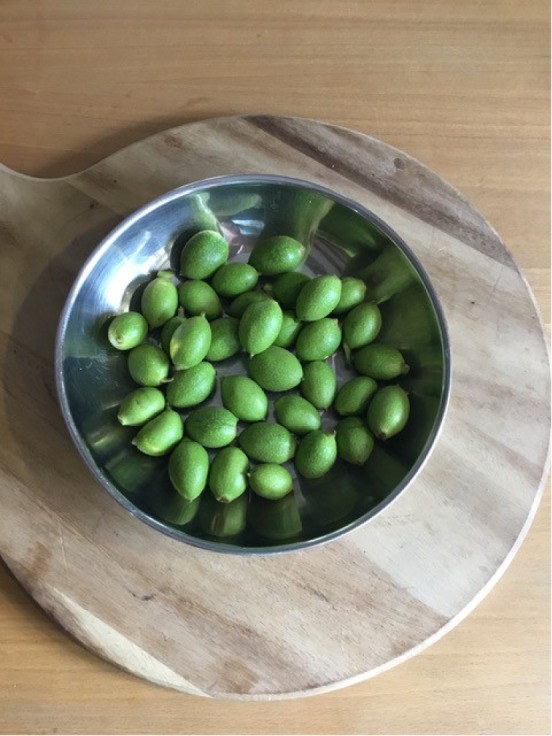
Fresh walnuts. (Photographed by the author.) (Online version in colour.)

Although I followed five different recipes for preserved walnuts in the reconstruction exercise, there were several fundamental steps that were common to all of them, which I shall now describe. I chose to undertake the reconstruction at my parents' home in Suffolk, given that they had a very generous neighbour who was willing to let me harvest walnuts from his tree. I picked the walnuts in June ‘around midsummer’ as instructed in the recipes. Having never seen a fresh walnut before, I was quite surprised at their appearance (figure 1): the bright green, smooth outer casing was very different from the wrinkled, gnarled walnuts familiar in the supermarket. Engaging with the recipes thus changed my idea of what walnuts actually are.

**Figure 2.**
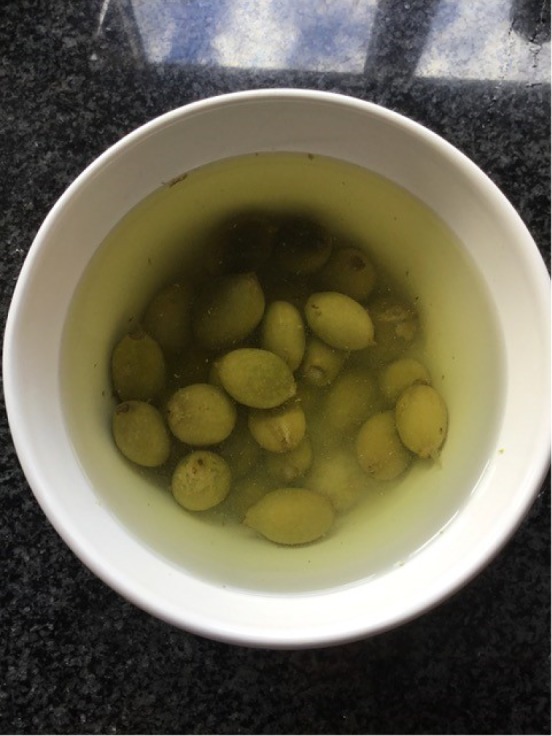
Soaking walnuts. (Photographed by the author.) (Online version in colour.)

**Figure 3.**
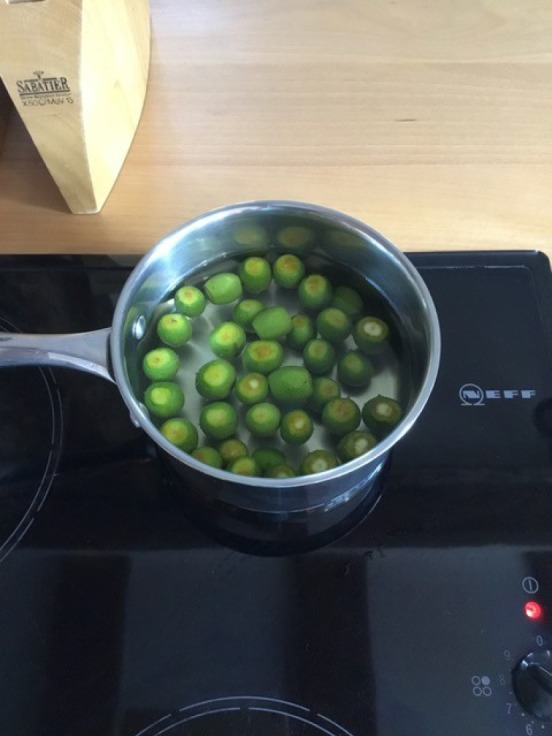
Boiling walnuts. (Photographed by the author.) (Online version in colour.)

**Figure 4.**
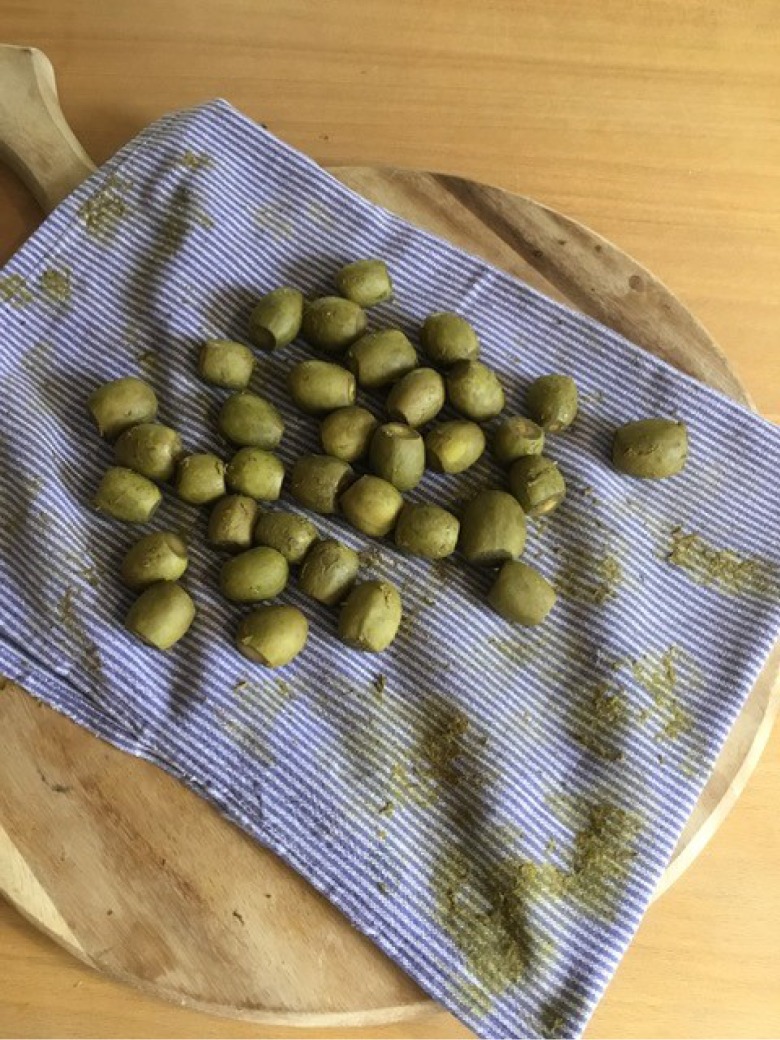
Drying walnuts. (Photographed by the author.) (Online version in colour.)

I began by soaking the walnuts, sometimes overnight, sometimes for several days, either in water or in a mixture of water and salt. Often the quantities of water and salt were not indicated in the recipes, so I decided to use enough water to cover the walnuts and a handful of salt.^30^ The vessel was also not specified, so I used a readily available ceramic bowl (figure 2).^31^ Then I rinsed the walnuts placed in a colander under running water. One imagines early modern householders would have used rainwater or water from a well or pump. Although I have not come across mention of a colander as such in the recipe books, there are references made to a ‘strainer’, so there must have been a similar sort of utensil in use in the early modern period.^32^ Next, the walnuts were boiled (figure 3), in some recipes multiple times. I used an electric hob and a metal saucepan to do this, not an open fire as early modern women would have done, purely for reasons of safety and accessibility. The smell of the boiling walnuts was very distinctive, like very strong tea, and hung around the kitchen for several hours afterwards. After boiling the walnuts, they were dried; I used an old tea towel to do this (figure 4). This part of the recipe was very messy: the outer green skin of the walnuts rubbed off after the boiling process, staining my hands (and the tea towel).

**Figure 5.**
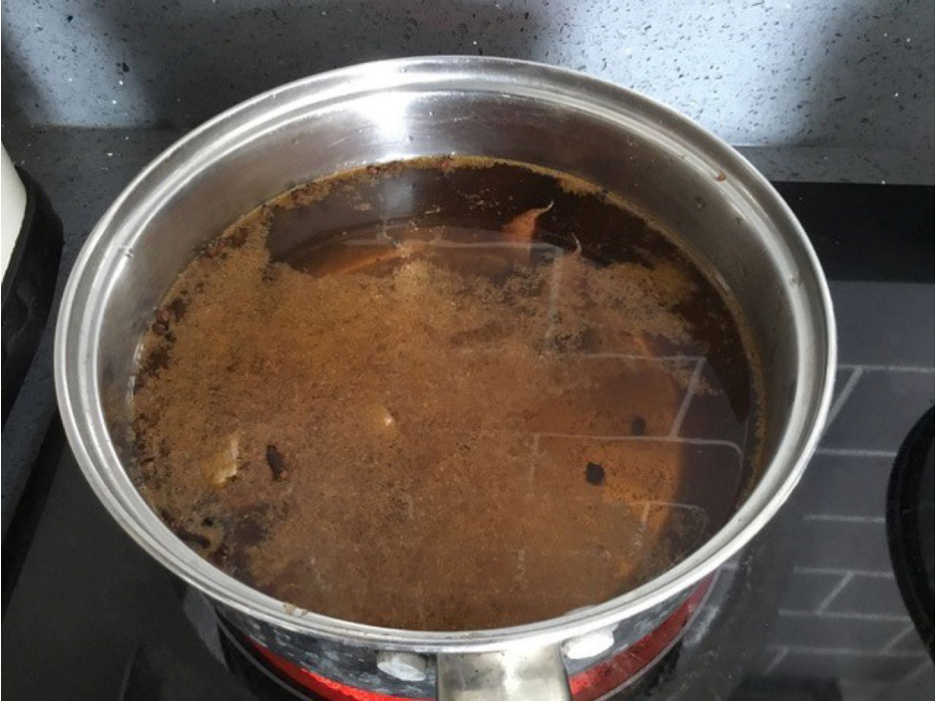
Pickling liquor. (Photographed by the author.) (Online version in colour.)

**Figure 6.**
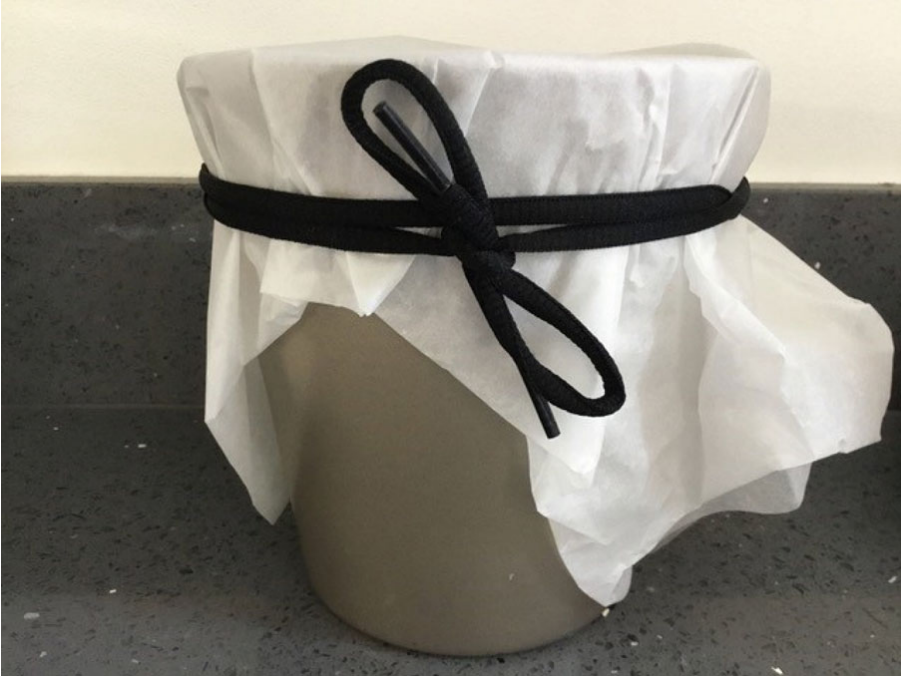
Sealing the vessel. (Photographed by the author.) (Online version in colour.)

I then made a pickling liquor (figure 5), often with white wine vinegar, several spices and sometimes sugar. This again produced a very pungent smell, owing to the vinegar, that made my eyes water. The final step was to add the prepared walnuts to the pickling liquor and seal the vessel. The recipes often indicated that this had to be done by tying a piece of leather around the top, presumably around the lid of the pot, although this was not specified.^33^ For many of the pots and bowls I used to store the walnuts I did not have accompanying lids, so I used folded up pieces of greaseproof paper to cover them, and some string (actually a spare shoelace) to tie around the top (figure 6).

Preserving the walnuts was not easy, and it was not particularly enjoyable: this was messy, smelly and laborious work. I found myself having to interpret lots of the instructions and trying to make ‘educated guesses’ as to what the early modern housewife would have done. Reconstruction not only brought me closer to the sensory experiences of the early modern housewife in preserving activities, but it was also invaluable in highlighting the domestic skills and tacit knowledge required for this process.

I identified three different forms of tacit domestic knowledge through reconstruction: *assumed* domestic knowledge; *thrift-orientated* domestic knowledge; and *experiential* domestic knowledge. *Assumed* domestic knowledge was indicated through references to a body of knowledge that the writer believed the reader to be familiar with. This was made especially evident in relation to the quality of types of ingredients used in preserving. In the reconstruction exercise, Bridget Hyde's recipe ‘To Pickle Walnuts’ called for ‘the best wine vinegar’ to be used to make the pickle, and it was assumed that the reader knew what this meant.^34^ During reconstruction, I found it challenging to know what comprised ‘best’: was this the most expensive? Or did it mean that the vinegar had to have a certain degree of acidity, or be made from a particular type of white wine? Did it have to smell a certain way, or taste a certain way? I ended up using an organic brand of white wine vinegar, although more specific details would have helped clarify the author's intention. The early modern housewife would need to be able to judge the quality of ingredients, as well as knowing where to source them, as we have already seen.

Another example can be found in the recipe ‘To pickle greane walnutts’. The instruction was given to ‘boyle them till they are a little softish but not to be to soft’.^35^ No further details were given regarding how soft was ‘to soft’. I interpreted this instruction to mean soft enough to pass a sharp knife through easily, while ensuring that the walnuts still kept their shape and form. However, it is unclear as to whether this is what the recipe writer actually intended.

*Thrift-orientated* domestic knowledge is indicated through a deliberate vagueness by the writer in reference to the quantities of ingredients required for the recipes. This gave the cook the freedom to use what they had available, thereby allowing them to maintain an ethos of ‘thriftiness’.^36^ I found that this form of domestic knowledge required judgement and understanding as to what would make a successful preserve.

The recipe ‘To pickle walnuts’ in MS.2990 instructs that, when making the pickle, the cook should ‘put in white pepper, ginger, Cloves & Mace of each a like quantity enough to make it strong of the spice’.^37^ This indicates that the senses needed to be utilized: smell and perhaps also taste, in order to ensure that the pickle was ‘strong’. During reconstruction, I did not have any white pepper and only had a little ground ginger, so I replaced the white pepper with black, put what ginger I had into the mixture and added a teaspoon of each of the remaining spices. As this resulted in the mixture smelling fairly pungent and tasting as such I believed it was the ‘correct’ amount. In this way I was inadvertently ‘thrifty’, using what I had available at the time. It did not seem to matter that I did not have equal amounts of all the spices.

*Experiential* domestic knowledge is a type of tacit knowledge that drew on a general domestic understanding and prior experience in cooking. There was some crossover with *assumed* domestic knowledge in that an assumption of relevant experience was clearly made. Bridget Parker's recipe book contains a recipe ‘To preserve walnuts physichaly’. After boiling the walnuts, the cook was instructed to ‘cut ym in ye sids wth a pen Knife to ye midle & stick half a clove in each of ym’.^38^

**Figure 7.**
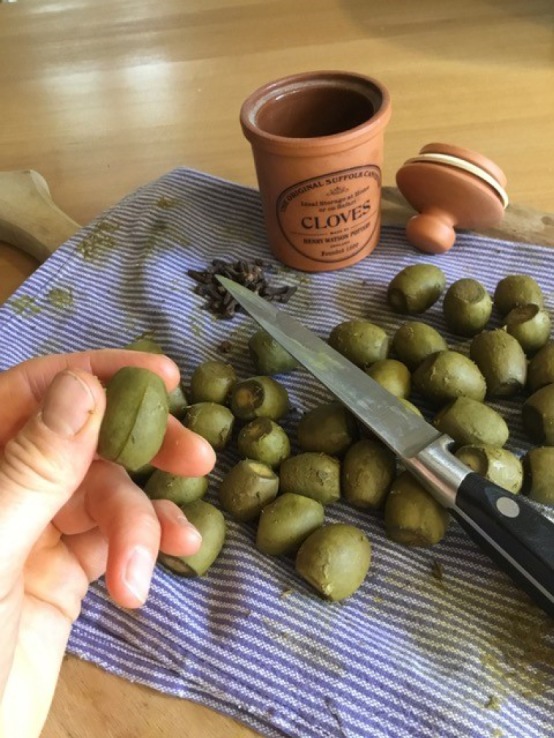
Cutting the walnuts. (Photographed by the author.) (Online version in colour.)

**Figure 8.**
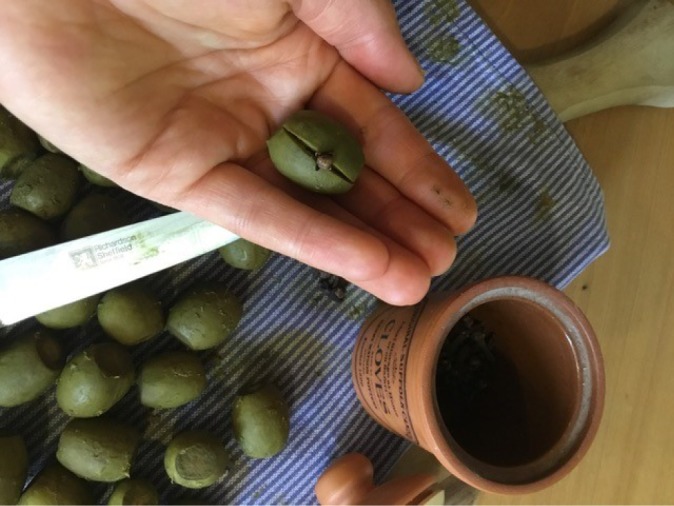
Inserting cloves. (Photographed by the author.) (Online version in colour.)

During reconstruction, I was initially unclear as to whether the cloves should be inserted vertically or horizontally into the slit made with the knife (figure 7). It was only after attempting to insert the cloves horizontally that I found this to be impossible, given the nail-like shape of the cloves with a sharp point at one end. I therefore ended up inserting the cloves vertically (figure 8). Had I experience in making this recipe, I would have known that this was the correct method.

## Preservation in context

Much effort was spent by early modern housewives compiling and keeping recipe books.^39^ Interestingly, Ann Blair speculates that the very nature of recipe books and ‘how-to books’ as the products of compilation has ‘deterred scholarly attention … until recently’.^40^ Historians have disparate views regarding the motivation behind recipe book compilation. Kristine Kowalchuk argues that this activity was an exercise in record keeping and was primarily performed in order to preserve in a material form the verbal culture of recipe creation.^41^ However, Lynette Hunter and Catherine Field have a slightly different view, positing that recipe books had more of a social function, particularly for women, nurturing a network of friends and acquaintances, and serving as a ‘leisure activity’.^42^ Michelle DiMeo and Sara Pennell similarly comment upon the ability of recipes to provide opportunity for interaction with ‘other people …, times, places and cultures’.^43^ In this way, recipe book compilation might be seen as a form of escapism, ‘tak[ing] its makers far from the kitchen hearth – aesthetically …, geographically, socially and intellectually’.^44^

I argue that there are two main reasons behind recipe book compilation. Motivation in part came from the responsibility that housewives had to provide for the family and to ensure adequate food throughout the winter: this was their duty, and they needed to assimilate the relevant knowledge in order to carry out this task.^45^ The second reason is that recipe books gave housewives an opportunity for autonomy in the creation, ownership and exchange of ‘domestic knowledge’. They provide ample material evidence of highly active domestic networks that existed in the early modern period for the purposes of sharing and disseminating knowledge through the medium of recipes. This can be seen through the attribution of certain recipes to particular individuals: ‘To Pickle Radishes Mad^m^ Hunters’, for example.^46^

A sample of twelve manuscript recipe books from the Wellcome Library's extensive collection provides evidence of preserving techniques and is the focus of this section. The manuscripts were selected according to the date of compilation (all were initially compiled in the seventeenth century), and the presence of a range of food preservation recipes, including at least one for preserved walnuts. On average, at least half of the food recipes in each manuscript involve preservation in one form or another; the majority of these are for fruits and vegetables, although recipes to preserve meat and fish also feature. The manuscripts are of varying length and size, ranging from 16 to 93 leaves and from folio to octavo size. Some of the recipe books are clearly ordered, with indexes and prescribed sections for food recipes and medicinal remedies, while others appear more haphazard, with recipes for medicines found in and among those for food and domestic tips and tricks.^47^

Regarding ownership, one recipe book is completely anonymous,^48^ whereas the others are all associated with particular individuals (all women). Very little is known about these women. We do not even know the first name of ‘Madam Carr’; the last leaf of the main text of MS.1511 simply states: ‘Madam Carrs Booke, Feb. 26^th^ 1681/2'.^49^ Information relating to the owners Bridget Parker, Elizabeth Godfrey, Mary Miller, Sarah Hudson and Elizabeth Jacob is similarly sparse, with only a date specified in the recipe books next to their name; this is assumed to be the date of compilation.^50^ The social standing of some of these women can be inferred. For example, Lady Ayscough, whose name is inscribed on the front flyleaf of MS.1026, was clearly a member of the gentry.^51^ A little more is known about Bridget Hyde, who compiled MS.2990. The daughter of Sir Thomas Hyde, Bridget married Peregrine Osborne, Duke of Leeds. Her recipe book was obtained from the Hornby Castle Library through Sotheby's auctioneers in 1930.^52^

**Figure 9.**
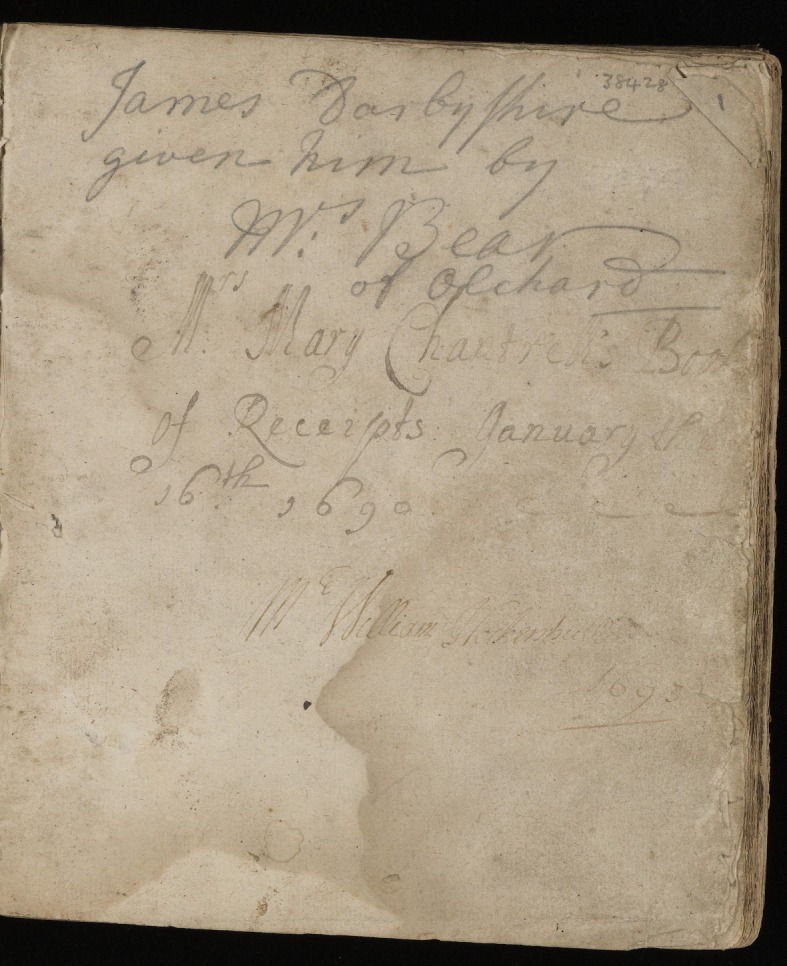
Mary Chantrell's recipe book, Wellcome Collection MS.1548, f. 1r. (Courtesy of the Wellcome Collection, London.) (Online version in colour.)

Mary Chantrell's name is written on the first leaf of her recipe book: ‘Mrs. Mary Chantrell's Book of Receipts January the 16^th^ 1690’ (figure 9). Below, in another hand, is written ‘Mr William Hockenhull 1693’. Above these, in pencil, by a nineteenth-century hand, is ‘James Darbyshire given him by Mrs Bear of Olchard’.^53^ This evidence of ownership, by both men and women, over the centuries gives us some sense of the value and durability of recipe books. Sara Pennell's in-depth study of Hannah Bisaker's recipe book (Hannah Buchanan after she married) highlights the inclusion of a list of names on one of the leaves, believed to be the children of Hannah and her husband, Michael.^54^ An entire page is dedicated to the names of Ann, Sarah, John, William, Edward and George, giving some indication of how central the manuscript was to the family.^55^

## The objects and tools of preservation

What was preserved when in the seventeenth century was strongly dictated by seasonality and accessibility. Most of the fruits mentioned in the Wellcome recipe books would have been locally grown in England, for example ‘Gosberys’, ‘pipins’, ‘Damsons’ and ‘plums’.^56^ However, some are more exotic, such as oranges and lemons, and had to be imported. As Stobart has shown, importing food in the early modern period was more common than historians have tended to appreciate, especially among the gentry.^57^ Vegetables are prevalent in the preserving recipes, with instructions for pickling the likes of mushrooms, artichokes and radishes.^58^ Given that animals were often slaughtered at the start of the winter so that they would not have to be fed valuable food during times of scarcity, there are also recipes to salt, pot and dry meat.^59^ Perhaps optimistically, recipes ‘To keepe Venison a year’ are not uncommon.^60^ Even flowers and herbs could be the focus of preservation, with recipes ‘To Candy flowers of Roses violets Cowslips Borage’.^61^ These confections often had medicinal applications as Ivan Day has demonstrated.^62^ Flowers might also feature in preserved drinks, for instance ‘Cowslip Wine’, while recipes to preserve herbs in some form include those for ‘Benjamin water’ and ‘To Candy Angellico’.^63^ As Wendy Wall notes, distilled ‘waters’ had a role in medicines, as well as being used in food seasonings and perfumes.^64^ Despite preserved walnuts being the main focus of the reconstruction exercise, nuts in general are not common in the recipes examined. Walnuts appear to be the only nuts preserved, aside from almonds in the form of ‘almond butter’.^65^

**Figure 10.**
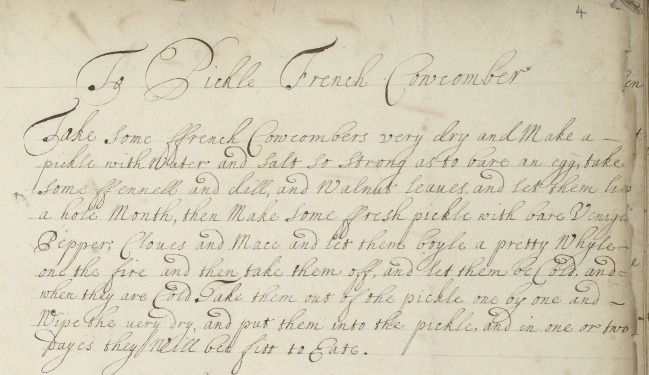
A recipe ‘To Pickle French Cowcomber’, Wellcome Collection MS.2990, f. 4r. (Courtesy of the Wellcome Collection, London.) (Online version in colour.)

Much can be learnt through textual analysis of the recipe books about the extent of domestic knowledge required for preservation. Typically, each recipe is written in prose as a single paragraph; there are no separate lists of ingredients or utensils as is the case in modern-day recipes. MS.2990 contains a recipe ‘To Pickle French Cowcomber’ (figure 10).^66^ An example of domestic knowledge can be found in this recipe, as the reader is instructed to ‘Make a pickle so strong as to bare an egg’.^67^ The reader therefore needed to know the correct amount of vinegar to add to the pickle in order to ‘bare’ an egg: that is, dissolve its shell without damaging the delicate inner membrane.

The need for domestic knowledge may also be appreciated in recipes involving the heating of sugar and the making of syrups. A recipe to preserve gooseberries begins: ‘Take 3 Quarters of a Pound of double refined Sugar beaten … Put to it halfe a Pinte of Water, boyle it & skimme it till it is Candy height’.^68^ Although ‘Candy height’ is not defined in the recipe, the early modern reader presumably understood the term.^69^ Sugar work features regularly in seventeenth-century recipe books as Jayne Archer and others have demonstrated, suggesting that it was frequently practised in the early modern kitchen.^70^ Without domestic sugar thermometers, the housewife would need to use her powers of close observation and associated knowledge to determine the correct point at which to stop heating the syrup. This had important implications as sugar was a valuable commodity and heating the syrup too much would lead to burning: an irreversible process that would result in wasting the whole mixture.^71^

Analysis of the recipe books demonstrates that the compiler typically had extensive experience in cooking, as recipes often cross-reference one another. In a recipe to make ‘Pippin Marmalett’, the reader was instructed to ‘breake [the pippins] as you doo yo^r^ Quince Marmalett’.^72^ Another example occurs in a recipe for ‘Elder Wine’, where the reader was advised that ‘the Berries must be boild in a large Earthen Pott in a Kettle of water as you doe for Jelly’.^73^ These recipes give some indication of the expected cooking repertoire of housewives in the early modern period.

Knowledge about ingredients was also essential, both where to obtain them and how to judge their quality. Recipes to preserve walnuts provide us with a useful example to demonstrate this point. The ingredients for this delicacy were extensive. Although the basic components of the pickling liquid were simple enough—usually white wine vinegar and sugar—a variety of herbs and spices were added, not all of which could be locally grown or easily sourced. These might comprise white pepper, ginger, mace, dill, cinnamon, mustard seeds, cloves, bay leaves or a combination of all of these. Most of the recipes contain at least three herbs or spices.^74^ Stobart's analysis of the ingredients found in medicinal recipes has demonstrated that many herbs and spices were either difficult to cultivate, meaning that they were purchased from a specialist supplier, or were grown in warmer climes and had to be imported. Exotic spices included allspice, ginger, mace, cinnamon and nutmeg, all of which regularly featured in the recipe books.^75^ Although rosemary and sage were plants of the Mediterranean, it was possible to grow them in English gardens.^76^ Understanding where to obtain such items was crucial knowledge for the housewife, and advice was sometimes imparted in the recipe books. In MS.2535, in reference to a purging medicine, the writer stated that ‘Tarter Emeticum – a gray Poweder’ could be purchased for 3.d ‘at y^e^ still in Walbrock’; presumably this was an apothecary, although no further details are provided.^77^ This example gives some sense of the extensive networks that the early modern housewife would need to utilize in order to preserve effectively.

Domestic knowledge involved not only recipes but also how to organize the people to make them. Some recipe books recorded large quantities of ingredients that were presumably too great for a single individual to manage. For example, a recipe ‘To make Chocolat’ called for ‘25 pound [11.34 kg] of Coconuts’ and ‘16 pound [17.26 kg] of shugar’, while a recipe to make ‘Walnut water’ suggested using ‘a Gallon of Brandy [4.55 litres] and 2 gallons [9.12 litres] of White-wine’.^78^ Another recipe ‘To make Orange watter’ required ‘aight quarts [9.12 litres] of Brandy and halfe a hundred of the best rough coated oranges’.^79^ Implicit in these recipes, then, was a communal enterprise that the housewife needed to manage. This responsibility was increased because many ingredients, such as alcohol and sugar, or imported items such as cacao nuts and oranges, were expensive, perhaps indicative of the trust that a family placed in their cook.

The temporalities of preservation deserve some comment here. The large quantities of foods preserved were intended to last many months, sometimes even years in the case of distilled flavoured waters. This implies that the makers believed in the durability of the household despite the constant threat of disease and economic turmoil; even if the cooks themselves did not live to eat the last of the pickled walnuts, members of their families would.

A variety of different utensils and equipment were required in order to preserve in the early modern kitchen and these ranged from simple everyday items to more specialized apparatus. In the Wellcome recipe books, references are made that suggest the extent of this ‘methodological’ domestic knowledge. In a recipe ‘To make Whit Quince Marmalade’, the cook needed to use ‘a little bagg of whit Cotton such as Jelly bagge is made off’.^80^ In this case, the reader was clearly expected to be familiar with the material used to make a ‘Jelly bagge’, and should therefore be able to fashion or source an appropriate substitute. In the recipes to preserve walnuts, various different types of vessel were required. These included, for example, ‘a new earthen pot well glazed’, a ‘skillet’, a ‘stone pott’ and a ‘kettle’.

Elizabeth Godfrey's recipe specified that the walnuts must be boiled in a brass pan, but when they stood in their syrup it should be in ‘Earthen pans’.^81^ In her work on the processing of linseed oil in the early modern period, the technical art historian Indra Kneepkens has shown that the choice of vessel was strongly influenced by the temperature required.^82^ Ceramic, bronze and copper vessels could withstand extremely high temperatures (over 500 °C). She also notes that the mention in several recipes of the ‘development of foam’ (which helped indicate the temperature of the contained liquid) was highly dependent on the cooking vessel used.^83^ In her own experiments, Kneepkens found that this ‘foam’ was visible when a glazed ceramic pot was used, but not when it was replaced by a glass laboratory beaker.^84^ The specification of particular vessels in the Wellcome recipe books suggests that early modern cooks understood these principles, and were thus able to control the heating of certain substances. While glazed ceramic pots might be used when substances needed to be heated to extremely high temperatures, earthen pans could be used in cases where the cook wished to temper the heat.

Recipes to make flavoured ‘waters’ required slightly more sophisticated apparatus in the form of an ‘allimbeck’ (alembic) to enable distillation. This piece of equipment had applications within the early modern practices of alchemy and chymistry.^85^ Likewise, a recipe ‘To make water of wallnutts’ instructs the reader to ‘still it in a stillatorie of glass’, though few details are given about the actual process.^86^ The importance of distillation is reflected in the growing popularity of ‘still-houses’ in the sixteenth and seventeenth centuries. These were separate buildings attached to the main house, created specifically for the purpose of distillation.^87^ Housewives might thus use quite specialized equipment, today associated with the history of science, and would also need the knowledge to operate it. Of course, not all early modern housewives had access to a still-house and it is worth noting that the lack of such apparatus did not necessarily restrict their preserving activities, as Pennell has shown. She argues that household inventories are a ‘poor index’ of food preservation practices, given that the dry-salting of meat and fish could occur in ‘nondescript tubs and chests’, while simple distilling was possible with ‘pans, gallipots and bottles’.^88^ However, housewives still needed the necessary ‘domestic knowledge’ to understand the modifications and substitutions that were needed in order to preserve effectively.

Further evidence to support the intuition required for the preserving process may be found in the adaptations made by housewives in utilizing everyday domestic items specifically for this purpose. In a recipe ‘To keep Damsins or Grapes fresh till Chrissmasse’, the reader was instructed to ‘Take a deep Earthen pot and a forked stick … hang your branches of Grapes or Damsins upon the stick’.^89^ Rather than buying a specific utensil or making something from scratch, the writer was looking to what was freely available in nature in order to carry out their task. In this way, not only was the writer being resourceful, but they were also being frugal by ‘making use’ of accessible materials. Werrett has argued that such ‘making use’ was typical of early modern experiments.^90^ This is implicit despite the relatively high social standing of some of the recipe book compilers, as we have already seen.^91^ Such ‘thriftiness’ was a reflection of the cultural and moral context rather than an indication of economic necessity.^92^ In a recipe ‘To preserve Apricocks’, once the fruit had been boiled in a basin, the reader was instructed to ‘Lay a Pillow upon it to keepe in ye heate soo let them stand all night’.^93^ Storing the fruit like this would ensure that it cooled slowly. In a recipe ‘To Pickle Cowcumbers’, ‘A Thread’ was used to tie the cucumbers together—something that would be commonly found in the domestic sphere for the purposes of needlework. The reader was later instructed to ‘wrap about [the Cowcumbers] a blanket, till it is cold’.^94^ Like the pillow used in the example earlier, this exemplifies both resourcefulness and understanding of the processes involved in food preservation. By wrapping up food, much like one's own body in the bedclothes at night, warmth could be maintained and the sudden cooling of substances prevented. In *Thrifty science*, Werrett has argued that early moderns often discussed material objects and their own bodies in similar language, and these examples would support the connection.

## Preservation as ‘experiment’

As Elaine Leong and Alisha Rankin have shown, some early modern domestic practices, such as the production of home medicinal remedies, can be interpreted as a form of ‘experiment’.^95^ They argue that ‘rather than focusing on “experiment” as a definitive and monolithic practice’ we should instead consider the ‘multiple strains of “experimental thinking” in various contexts’.^96^ The same occurs in the particular case of preserving recipes, which exhibit a number of experimental features. Preservation is ‘experimental’ both through its reliance on keen observation, and through the trying and testing of recipes, with an adjudication of which ones have succeeded, and which have not. These were important ingredients of experiment in more familiar settings such as the Royal Society, as Simon Schaffer and Steven Shapin have demonstrated, but they were also present in the domestic kitchen.^97^ The aim of this section is not to argue that, as a result of being experimental, domestic preservation was considered a form of natural philosophy by early moderns, but rather to situate food preservation within a broader context of early modern experimental practices.^98^

The importance of observation is evident in MS.1548, in a recipe for ‘Calves foot Gelly’. The recipe instructs the reader to ‘put it back again into ye bag *till it Look as clear as Christial* then putt it into Jelly glasses’.^99^ Likewise, a recipe ‘To Bottle Gooseberries’ required the cook to ‘let ’em boile gently till the Goosberries looke white in the Bottle, & beginn to Crack, *that you perceive that they begin to be a little tender*’.^100^ This excerpt is even more suggestive as it indicates that the housewife could tell when the gooseberries were ready using purely her powers of observation; no other senses were required. Pamela Smith makes the argument that early modern artisans utilized techniques of close observation in their workshops and, consequently, artisans had a more important part to play in experimental natural philosophy than they have previously received credit for.^101^ Likewise, Bert De Munck highlights the ‘valuable’ and ‘sophisticated’ skills of artisans and craftsmen, but suggests that the political and intellectual elites of the seventeenth and eighteenth centuries essentially reduced them to ‘being valuable in a narrow logic of productivity only’.^102^

**Figure 11.**
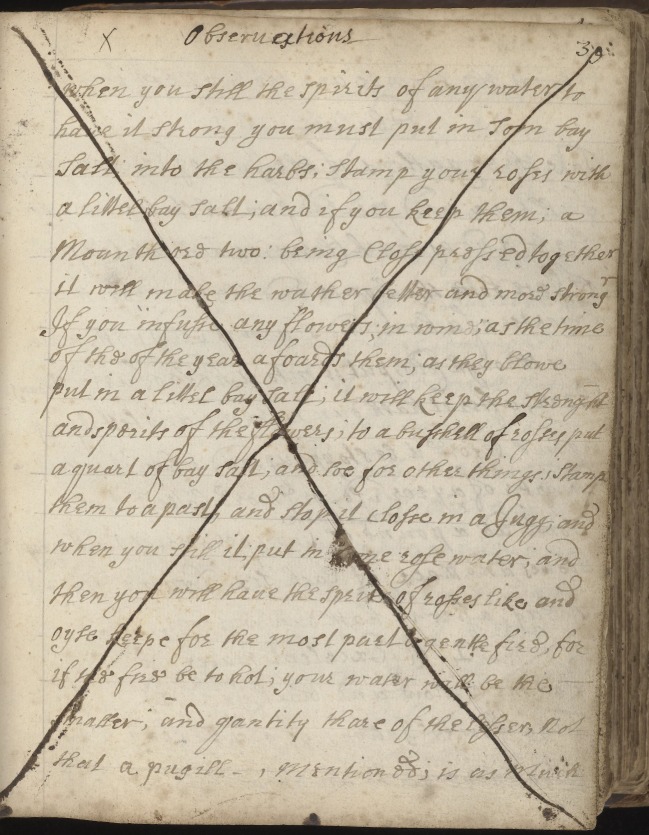
‘Observations’ on the process of ‘stilling’ flavoured waters. Wellcome Collection MS.3009, f. 329v. (Courtesy of the Wellcome Collection, London.) (Online version in colour.)

I argue that early modern food preservation also embodies this technique of close observation. Perhaps the most striking example to demonstrate this is a page from the manuscript recipe book attributed to Elizabeth Jacob. Here, a recipe ‘To make Mead’ is followed by a page entitled simply ‘Observations’ (figure 11), where the author gives advice related to the process of ‘stilling’ to produce various flavoured waters:

Observations

when you still the spirits of any water to

have it strong you must put in Torn bay

Salt into the harts; Stamp your roses with

a little bay salt; and if you keep them; a

Mounth ore two; being Close pressed together

it will make the watter better and more strong^103^

Interestingly this page, and many of those following it, have been crossed out. Although it is still possible to read these passages, one inference is that a subsequent owner of this manuscript disagreed with the accuracy of these ‘observations’. In Leong's study of Lady Anne Fanshawe's recipe book, she describes how Fanshawe marked out notable recipes with a cross alongside them, while recipes crossed out indicated a failure to meet expectations.^104^

The crossing out itself is further evidence of experimental practices. The extensive annotations, corrections, deletions and additions in recipe books are indicative of trying and testing recipes. Recipe books were very much works in progress: ‘living manuscripts’ that were constantly edited and changed over time.^105^ These annotations helped clarify and refine the cook's instructions, facilitating the re-creation and replication of these recipes, a key feature of experiment.^106^ As Kowalchuk states, ‘recipes and remedies were continuously added into the manuscript or annotated right in the kitchen’—in a similar way to the ‘scientific’ notebooks of the likes of Robert Boyle.^107^ Interestingly, Werrett notes that Boyle's work diaries ‘made no distinction between chymical, experimental, medical and culinary procedures’.^108^ This emphasizes further the need to consider these practices collectively and to resist the modern-day tendency to categorize, classify and isolate such activities.

**Figure 12.**
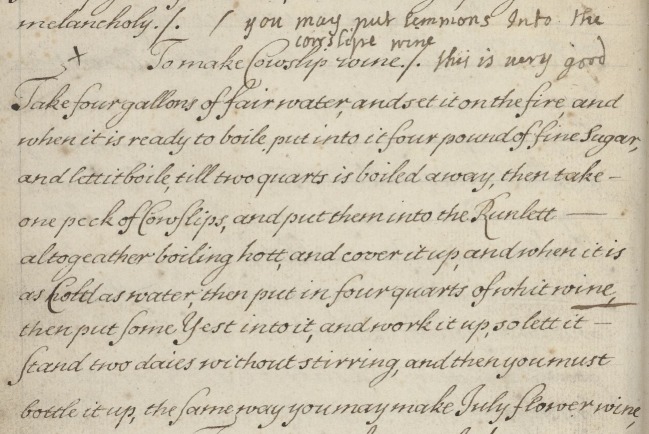
Recipe ‘To make Cowslip wine’, Wellcome Collection MS.3009, f. 335r. (Courtesy of the Wellcome Collection, London.) (Online version in colour.)

The link between the terms ‘experiment’ and ‘experience’ is evident in the recipe books, through the indication of experience in preserving.^109^ For example, the title of one recipe in MS.3009 is ‘To make Cowslip wine’, and next to this is the annotation that indicates one maker's experience: ‘this is very good’ (figure 12).^110^ Given that this comment is in a different hand, we can infer that it was written by a subsequent reader or owner of the recipe book, who had tried this recipe out for themselves. This is further suggested by the remark above the recipe, ‘you may put lemmons into the cowslip wine’.^111^ The indication of experience adds credence to the comments made and would be particularly persuasive to subsequent readers of this recipe. A final example, from MS.2990, is even more explicit in indicating the importance of prior experience in preserving. In a recipe ‘To Make Spirit of Rosses’, the author writes that ‘the stronger firer maketh the most oyle, a temperate fire is held to weaken the sweetes, as Expirience will show’.^112^

Another way to infer experience was through the provision of circumstantial details. As Schaffer and Shapin among others have shown, this was certainly the case for the Royal Society and can be seen in formalized publications such as *Philosophical Transactions*: it was one way of assuring readers that the experiments described resulted in the findings stipulated.^113^ This can also be demonstrated in the recipe books. In a recipe for ‘Marmalet of Oranges’, the reader is told that ‘if you keep out some of the sugar and stow it in the boyling it will jelly the sooner’.^114^ Not only was the writer providing useful additional information, but they were hinting at their extensive experience and promoting themselves as a trusted and educated source.

These examples closely link to another experimental feature of recipe books: that of claims and efficacy statements. Often penned at the end of the recipe, and ranging from a simple *probatum est* (‘it has been proved’) to more detailed statements explaining the virtues of the recipe in question, efficacy phrases were designed to instil trust in the reader and encourage them to believe that the recipe did what it claimed.^115^ Francisco Alonso-Almeida has explored the use of *probatum est* in early modern manuscript recipe books and has speculated about the potential meanings and functions of this phrase. *Probatum est* may have acted to increase the interpersonal distance between the reader and writer, through the use of the academically superior Latin language. Alternatively, it may have worked to foster associations with practices of religion and magic, as both were strongly affiliated with the use of Latin. Finally, Alonso-Almeida theorizes that the use of Latin could reflect ‘professional approval’, hence heightening confidence in the validity of the recipe in question.^116^

Discussion about efficacy statements should be considered within the broader context of early modern note-taking and manuscript annotations. In her study of early modern note-taking, Ann Blair highlights the fact that notes were valued both by those who took them and by others ‘who hoped to put them to use’.^117^ We can therefore appreciate that annotations in recipe books were not simply absent-minded doodles but carefully considered words of advice, guidance and criticism, which were intended to be read.

Some efficacy statements in the recipe books relate to the quality of the final product. For example, a recipe ‘To Make Wormewood wine’ ends with the proclamation that it ‘will make as good wormwood wine as ever was drunke’.^118^ Other efficacy statements relate more specifically to the recipe's ability to preserve food effectively. In a recipe ‘To keep Damsons or graps’, the writer uses clay in the preservation process; they claim that: ‘you may keep oranges or lemons all the yeare & ye grapes and damsons fresh until xmas if you stick a rose in clay in this pot you shall find it fresh at Chrismas’.^119^ Efficacy statements are particularly numerous in those recipes with medical applications. In a receipt ‘For a Cancer in y^e^ Breast’, it is stated that ‘this Dissolved a Cancer in Mrs Hartley's Breast which was designed to have beene cut of by Mr Hobbs’.^120^ In situations where more conventional medical practitioners were difficult to access, through restrictions related to money or locality, for example, it is easy to see how such claims related to domestic remedies would have made for welcome reading. Leong and Pennell's work on the medical recipes in early modern manuscript recipe books highlights the value of medical recipes and suggests that they can be seen as ‘analogous to particular forms of early modern financial transaction … in that their realisable value was tied up with the trustworthiness of the relationship on which the exchange was based’.^121^

**Figure 13.**
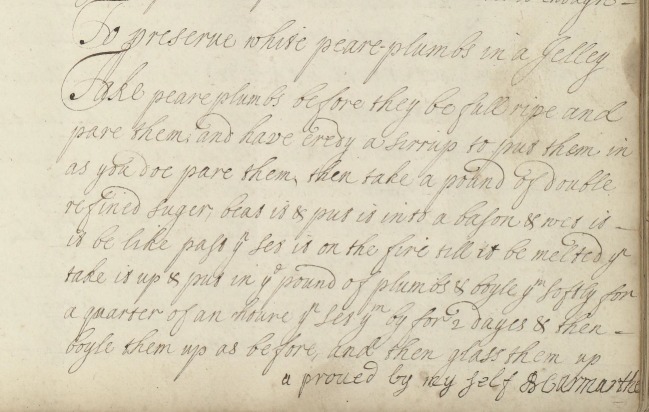
Recipe ‘To preserve white peare plumbs in a Jelley’, Wellcome Collection MS.2990, f. 36r. (Courtesy of the Wellcome Collection, London.) (Online version in colour.)

A recipe ‘To preserve white peare plumbs in a Jelley’ ends with an added line in a different hand stating ‘aproved by my self B Carmarthe’ (figure 13).^122^ This provides unequivocal evidence of the trying and testing of early modern preserving recipes. Seth LeJacq argues that the use of efficacy statements introduces us to the crucial point of proof as something closely dictated by personal experience.^123^ This links to Steven Shapin's work on the importance of ‘testimony’ in early modern natural philosophy. He emphasizes that ‘testimony … was crucial’ in the ‘natural history sciences’ and ‘experimental science’ of the early modern period.^124^ This discussion helps us to draw parallels between early modern cooking practices and the activities of natural philosophical institutions such as the Royal Society.

In the same way that the experiments of the Royal Society reflect the social context into which they were born, so the recipe books mirror the social context in which they were generated. Shapin and Schaffer argued that, for knowledge to be empirically based, it had to be witnessed, and one of the most straightforward ways to achieve this was by performing experiments in a social space.^125^ The Royal Society provided the perfect forum for this kind of activity. In the domestic kitchen, although the practice of cooking might be witnessed by various members of the household, it was seen as an ordinary everyday task and was therefore unlikely to receive undue attention. In contrast, the experiments of the Royal Society were deliberately performative.^126^ Although the cooking process might not be consciously witnessed, the act of tasting worked to substantiate the claims made in the recipes: the proof really was in the pudding.

The affiliation of some recipes with particular individuals acted to provide personal assurance regarding the success of the recipe in question. Even more familiar associations, such as a recipe from a close family member, would impart confidence in the reader: an ‘ordinary pomatum Ant harrisons’, for example.^127^ A sense of authority is particularly evident in those medical recipes associated with physicians or surgeons, such as the recipe in MS.2535 for ‘Docter Cox's Receipt for y^e^ Scurvy’.^128^ Other recipes were attributed to employers or individuals of high social standing: ‘My Lady Cheeks way how to Dry Plums’, or ‘To preserve whole oranges in jellyes my Lady Dukes way’.^129^ Here we can see the writer emphasizing that these recipes were from a trusted source, not a charlatan or a pretender.

## Conclusion

This paper has attempted to demonstrate that early modern preserving practices required skill, observation and an extensive knowledge base. Far from providing a step-by-step guide of how to preserve, manuscript recipe books instead indicate the need for a significant amount of tacit knowledge. I have argued that preservation was ‘experimental’ and an epistemic practice that might be considered under the umbrella of early modern natural philosophy. The social function of recipe books was integral to their compilation. They provided women with autonomy and purpose outside the household, considered to be ‘first and last, a domestic and feminine space’, through exchanging, obtaining and sharing preserving recipes in their communities and beyond.^130^

We have considered how textual analysis of early modern recipe books can indicate the extensive knowledge employed when preserving, while the technique of reconstruction can help recover the details of the ‘tacit’—what I have called ‘domestic’—knowledge required. This approach has helped establish the meaning and importance of preserving to early moderns. Given the restricted length of this paper, there is clearly more research to be done. Related further study might include examination of the acquisition of tacit domestic knowledge. In what ways was domestic knowledge learnt and passed on? How did the home environment foster this form of education? What was the significance of domestic networks in the process? The links between the practices associated with the early modern kitchen and those of the Royal Society might also be explored in greater depth.

The broader implications of my argument are that it helps us to establish the early modern kitchen as a space for significant knowledge-making, and encourages us to recalibrate our understanding of the fluidity of experimental practices in the early modern period, fighting against the compartmentalization of particular disciplines and activities. From a feminist perspective, rather than cherry-picking exceptional individual women, we should examine the often mundane, humdrum activities of ordinary seventeenth-century housewives, and broaden our definition of natural philosophy in the early modern period. We need to continue to look outside conventional academic institutions like the Royal Society and into the private sphere of the home. Recipe books are useful in providing us with a window through which to view this distinctly domestic space.

## Footnotes

1 Gervase Markham, *Countrey contentments, or The English Huswife* (London, 1615), title page, http://eebo.chadwyck.com/ (accessed 23 March 2019).

2 See for example: Sara Pennell, *The birth of the English kitchen, 1600–1850* (Bloomsbury Academic, London, 2017); Joan Thirsk, *Food in early modern England: phases, fads, fashions, 1500–1760* (Hambledon Continuum, London, 2007).

3 Antonia Fraser, *The weaker vessel: woman's lot in seventeenth-century England* (Weidenfeld & Nicholson, London, 2011), p. ix.

4 For example: Elaine Leong and Alisha Rankin, ‘Testing drugs and trying cures: experiment and medicine in medieval and early modern Europe’, *B. Hist. Med.* **91**(2), 157–182 (2017), at p. 162; Fraser, *op. cit.* (note 3); Amanda Herbert, *Female alliances: gender, identity, and friendship in early modern Britain* (Yale University Press, New Haven, 2014), pp. 78–116 (‘Cooperative labor: making alliances through women's recipes and domestic production’); Michelle DiMeo, ‘“Such a sister became such a brother”: Lady Ranelagh's influence on Robert Boyle’, *Intellectual Hist. Rev.* **25**(1), 21–36 (2015); Sara Pennell, ‘Consumption and consumerism in early modern England’, *Hist. J.* **42**(2), 549–564 (1999); Lynette Hunter, ‘Women and domestic medicine’, in *Women, science and medicine 1500–1700* (ed. Lynette Hunter and Sarah Hutton), pp. 89–107 (Sutton Publishing, Stroud, 1997); John Christie, ‘Feminism and the history of science’, in *Companion to the history of modern science* (ed. R. C. Olby, G. N. Cantor, J. R. R. Christie and M. J. S. Hodge), pp. 100–109 (Routledge, London, 1990); Margaret Rossiter, ‘A twisted tale: women in the physical sciences in the nineteenth and twentieth centuries’, in *The Cambridge history of science vol. 5: the modern physical and mathematical sciences* (ed. Mary Jo Nye), pp. 54–71 (Cambridge University Press, Cambridge, 2002).

5 Stuart Thorne, *The history of food preservation* (Parthenon Publishing, Kirkby Lonsdale, 1986), p. 14; Kristine Kowalchuk (ed.), *Preserving on paper: seventeenth-century Englishwomen's receipt books* (University of Toronto Press, Toronto, 2017), p. 9. I define ‘housewife’ as a married woman whose principal occupation was to manage her own household.

6 Markham, *op. cit.* (note 1).

7 C. Anne Wilson, ‘Preserving food to preserve life: the response to glut and famine from early times to the end of the Middle Ages’, in *Waste not, want not: food preservation in Britain* (ed. C. Anne Wilson), pp. 5–31 (Edinburgh University Press, Edinburgh, 1991); Peter Brears, ‘Pots for potting: English pottery and its role in food preservation in the post-mediaeval period’, in *ibid.*, pp. 32–65; Thirsk, *op. cit.* (note 2).

8 Wendy Wall, *Recipes for thought: knowledge and taste in the early modern English kitchen* (University of Pennsylvania Press, Pennsylvania, 2016), p. 169. Although botanically, cucumbers are defined as a fruit, in a culinary sense, they are defined as a vegetable. See John Staughton, ‘Organic facts’, https://www.organicfacts.net/cucumber-fruit-vegetable.html (accessed 31 August 2018).

9 Anne Stobart, *Household medicine in seventeenth-century England* (Bloomsbury Academic, London, 2016), p. 118. See also Thirsk, *op. cit.* (note 2).

10 Thirsk, *op. cit.* (note 2), p. 50.

11 See Marieke M. A. Hendriksen, *Elegant anatomy: the eighteenth-century Leiden anatomical collections* (Brill, Leiden and Boston, 2015); Marieke M. A. Hendriksen, ‘Anatomical mercury: changing understandings of quicksilver, blood, and the lymphatic system, 1650–1800’, *J. Hist. Med. Allied Sci.* **70**(4), 516–548 (2015); Rina Knoeff, ‘Touching anatomy: on the handling of preparations in the anatomical cabinets of Frederik Ruysch (1638–1731)’, *Stud. Hist. Philos. Biol. Biomed. Sci.* **49**, 32–44 (2014).

12 Daniel Margócsy, *Commercial visions: science, trade, and visual culture in the Dutch Golden Age* (University of Chicago Press, Chicago, 2014), p. 113.

13 ‘Tacit knowledge’ is defined by the sociologist of science Harry Collins as ‘knowledge that is not explicated’. See Harry Collins, *Tacit and explicit knowledge* (University of Chicago Press, Chicago, 2013), p. 1.

14 Hjalmar Fors, Lawrence M. Principe and H. Otto Sibum, ‘From the library to the laboratory and back again: experiment as a tool for historians of science’, *Ambix* **63**(2), 85–97 (2016).

15 Elaine Leong and Sara Pennell, ‘Recipe collections and the currency of medical knowledge in the early modern “medical marketplace”’, in *Medicine and the market in England and its colonies* (ed. Mark Jenner and Patrick Wallis), pp. 133–152 (Palgrave Macmillan, Basingstoke and New York, 2007).

16 See note 24 and associated text for further discussion.

17 In order to avoid anachronism, the term ‘natural philosophy’ will be used rather than ‘science’ throughout this paper. See Peter Dear, ‘What is the history of science the history *of*?’, *Isis*, **96**, 391–393 (https://doi.org/10.1086/447747)

18 Fors *et al.*, *op. cit.* (note 14), p. 89.

19 Thijs Hagendijk, ‘Learning a craft from books: historical re-enactment of functional reading in gold- and silversmithing’, *Nuncius* **33**(2), 198–235 (2018), at p. 212.

20 *Ibid.*. See full article for further details on each definitive methodological approach mentioned here.

21 Pamela Smith, ‘Historians in the laboratory: reconstruction of Renaissance art and technology in the making and knowing project’, *Art Hist.* **39**(2), 210–233 (2016), at p. 220.

22 *Ibid.*, p. 215.

23 Klaus Staubermann (ed.), *Reconstructions: recreating science and technology of the past* (NMS Enterprises, Edinburgh, 2011).

24 See: ‘The recipes project: food, magic, art, science and medicine’, https://recipes.hypotheses.org; ‘EMROC: early modern recipes online collective’, https://emroc.hypotheses.org; ‘Cooking in the archives: updating early modern recipes (1600–1800) in a modern kitchen’, https://rarecooking.com (all accessed 21 May 2018).

25 Thirsk, *op. cit.* (note 2), p. 77.

26 Elizabeth Jacob (& others) Recipe Book, 1654–c.1685, MS.3009, Wellcome Library, London.

27 Ann M. Blair, *Too much to know: managing scholarly information before the modern age* (Yale University Press, New Haven, 2010), p. 46.

28 For further explanation of Foucault's text, see Garry Gutting, *Michel Foucault's archaeology of reason* (Cambridge University Press, Cambridge, 2010), p. 142. For a contemporary example, see William Coles, *Adam in Eden, or, Natures paradise* (London, 1657), p. 3. In a section on ‘The Signatures and Vertues’, Coles describes how ‘*Wall-nuts* have the perfect Signature of the Head’ and ‘therefore salt made of those husks or barks, are exceeding good for wounds in the head’ and ‘it is very profitable for the Brain’.

29 Michel Foucault, *The order of things: an archaeology of the human sciences* (ed. R. D. Laing) (Pantheon Books, New York, 1970), p. 17.

30 For example, Bridget Hyde Recipe Book, 1676–1690, MS.2990, f. 48v, Wellcome Library, London; Elizabeth Godfrey Recipe Book, 1686, MS.2535, unpaginated, Wellcome Library, London.

31 Indeed, the decision to use a ceramic bowl may well represent my own tacit knowledge in the understanding that a glazed surface is resistant to salt solutions.

32 Mrs Carr Recipe Book, 1682, MS.1511, f. 26r, Wellcome Library, London. In a recipe ‘To Make Whit Quince Marmalade’, the cook was instructed to ‘straine through a Coars strainer’.

33 For example, Hyde, *op. cit.* (note 30), f. 48v.

34 *Ibid*.

35 Godfrey, *op. cit.* (note 30), unpaginated.

36 ‘Thrift’ is defined by Werrett as ‘the pursuit of a sensible balance between buying new and using old things, between spending and saving and making the most of the resources you already had’. Simon Werrett, *Thrifty science: making the most of materials in the history of experiment* (University of Chicago Press, Chicago and London, 2019), p. 9.

37 Hyde, *op. cit.* (note 30), f. 48v.

38 Bridget Parker Recipe Book, 1663, MS.3768, f. 30v, Wellcome Library, London.

39 Herbert, *op. cit.* (n. 4), p. 113. See also Pamela H. Smith, ‘Why write a book? From lived experience to written word in early modern Europe’, *B. German Hist. Inst.* **47**, 25–50 (2010).

40 Blair, *op. cit.* (note 27), p. 175.

41 Kowalchuk, *op. cit.* (note 5), p. 43.

42 Hunter, *op. cit.* (note 4), pp. 102–103. See also Catherine Field, ‘“Many hands hands”: writing the self in early modern women's recipe books’, in *Genre and women's life writing in early modern England* (ed. Michelle M. Dowd and Julie A. Eckerle), pp. 49–64 (Routledge, London, 2007).

43 Michelle DiMeo and Sara Pennell, ‘Introduction’, in *Reading and writing recipe books 1550–1800* (ed. Michelle DiMeo and Sara Pennell), pp. 1–22 (Manchester University Press, Manchester and New York, 2013), p. 13.

44 *Ibid*.

45 Hunter, *op. cit.* (note 4), p. 96.

46 Jacob, *op. cit.* (note 26), f. 215r.

47 For example, *ibid.*; and Lady Ayscough's Recipe Book, 1692, MS.1026, Wellcome Library, London.

48 Anonymous Recipe Book, 1575–1663, MS.6812, Wellcome Library, London.

49 Carr, *op. cit*. (note 32), f. 71v.

50 Parker, *op. cit.* (note 38); Godfrey, *op. cit.* (note 30); Mary Miller Recipe Book, 1660, MS.3547, Wellcome Library, London; Sarah Hudson Recipe Book, 1678, MS.2954, Wellcome Library, London; Jacob, *op. cit.* (note 26).

51 Ayscough, *op. cit.* (note 47).

52 Wellcome Library, ‘Bridget Hyde’, https://search.wellcomelibrary.org/iii/encore/record/C__Rb1924808__Sms.2990__Orightresult__U__X3?lang=eng&suite=cobalt&openHierarchy=true (accessed 22 August 2018).

53 Mary Chantrell Recipe Book, 1690, MS.1548, f. 1r, Wellcome Library, London.

54 Sara Pennell, ‘Making livings, lives and archives: tales of four eighteenth-century recipe books’, in DiMeo and Pennell, *op. cit.* (note 43), pp. 225–246, at pp. 228–9.

55 Hannah Bisaker Recipe Book, 1692, MS.1176, unpaginated, Wellcome Library, London. Further details about these women owners lie outside the scope of this essay, although it would make for an interesting study.

56 For example, see Chantrell, *op. cit.* (note 53), unpaginated.

57 Stobart, *op. cit.* (note 9), p. 61.

58 Ayscough, *op. cit.* (note 47), f. 137r; Hyde, *op. cit.* (note 30), f. 47r; Jacob, *op. cit.* (note 26), f. 215r.

59 For examples, see Godfrey, *op. cit.* (note 30), unpaginated; Jacob, *op. cit.* (note 26), f. 317v. See also Thorne, *op. cit.* (note 5), p. 14.

60 Carr, *op. cit.* (note 32), f. 8r.

61 Hyde *op. cit.* (note 30), f. 54v.

62 Ivan Day, ‘The art of confectionery’, https://www.historicfood.com/The%20Art%20of%20Confectionery.pdf (accessed 29 July 2018).

63 Anon., *op. cit.* (note 48), f. 85r; Carr, *op. cit.* (note 32), ff. 26v and 23v.

64 Wall, *op. cit.* (note 8), p. 187.

65 For example, see Godfrey, *op. cit.* (note 30), unpaginated.

66 Hyde, *op. cit.* (note 30), f. 4r.

67 *Ibid*.

68 Mary Perrot Recipe Book, 1695, MS.3834, f. 30r, Wellcome Library, London.

69 It is notable that today there are six stages of cooking sugar syrup: at the ‘soft-ball’ stage (112–116 °C), the syrup comes together to form ‘a soft clump with a bit of height to it’. Randal Oulton, ‘Cook's info’, https://www.cooksinfo.com/soft-ball-stage (accessed 8 July 2018).

70 For further reading on early modern sugar work, see, for example, Jayne Elisabeth Archer, ‘Women and chymistry in early modern England: the manuscript receipt book (c. 1616) of Sarah Wigges’, in *Gender and scientific discourse in early modern culture* (ed. Kathleen Long), pp. 191–216 (Ashgate Publishing, Farnham and Burlington, 2010); Sandra Jergensen, ‘Liquorice: “the spoonful of sugar that helps the medicine go down”’, *The recipes project*, 9 May 2013, https://recipes.hypotheses.org/1295 (accessed 25 March 2019).

71 Jon Stobart, *Sugar and spice: grocers and groceries in provincial England, 1650–1830* (Oxford University Press, Oxford, 2013), p. 25.

72 Miller, *op. cit.* (note 50), f. 10v.

73 Perrot, *op. cit.* (note 68), f. 30v.

74 See the appendix for transcribed preserved walnut recipes.

75 Stobart, *op. cit.* (note 9), p. 92.

76 *Ibid*., p. 90.

77 Godfrey, *op. cit.* (note 30), f. 115r. ‘Tartar emeticum’ was an antimony potassium tartrate, widely used as diaphoretic and emetic in the eighteenth and nineteenth centuries. See John S. Haller, ‘The use and abuse of tartar emetic’, *B. Hist. Med.* **49**(2), 235–257 (1975), at p. 237.

78 ‘Coconuts’ refers to cacao nuts, the basis of chocolate. Godfrey, *op. cit.* (note 30), unpaginated; Miller, *op. cit.* (note 50), f. 67r.

79 Anon., *op. cit.* (note 48), f. 77v.

80 Carr, *op. cit.* (note 32), f. 26r.

81 Godfrey, *op. cit.* (note 30), unpaginated.

82 Indra Kneepkens, ‘Boiling hot oil: on the assessment of temperature in late medieval processing of linseed oil’, *The recipes project*, 16 August 2018, https://recipes.hypotheses.org/11275 (accessed 1 April 2019).

83 *Ibid*.

84 *Ibid*.

85 Godfrey, *op. cit.* (note 30), unpaginated; Miller, *op. cit.* (note 50), f. 39r. An ‘alembic’ is defined in the *Oxford English Dictionary* as ‘a distilling apparatus, now obsolete, consisting of a gourd-shaped container and a cap with a long beak for conveying the products to a receiver’, https://en.oxforddictionaries.com/definition/alembic (accessed 28 June 2018).

86 Jacob, *op. cit.* (note 26), f. 273r.

87 Stobart, *op. cit.* (note 9), p. 118.

88 Pennell, *op. cit.* (note 2), p. 80.

89 Hyde, *op. cit.* (note 30), f. 30r.

90 Werrett, *op. cit.* (note 36), p. 34.

91 See the section above on ‘Preservation in context’.

92 Werrett, *op. cit.* (note 36), p. 34.

93 Miller, *op. cit.* (note 50), f. 12r.

94 Jacob, *op. cit.* (note 26), f. 231r.

95 Leong and Rankin, *op. cit.* (note 4), p. 158.

96 *Ibid*., p. 181.

97 On early modern experiment, see for example Simon Schaffer and Steven Shapin, *Leviathan and the air pump: Hobbes, Boyle, and the experimental life* (Princeton University Press, Princeton and Oxford, 1985); DiMeo and Pennell, ‘Introduction’, *op. cit.* (note 43); Leong and Rankin, *op. cit.* (note 4).

98 For further reading, see Werrett, *op. cit.* (note 36), pp. 54–56. Also Pamela Smith, *The body of the artisan: art and experience in the Scientific Revolution* (University of Chicago Press, Chicago, 2004); William Eamon, *Science and the secrets of nature: books of secrets in medieval and early modern culture* (Princeton University Press, Princeton, 1994).

99 Perrot, *op. cit.* (note 68), f. 32v, emphasis added.

100 *Ibid*., f. 31r, emphasis added.

101 Smith, *op. cit.* (note 98), p. 99.

102 Bert De Munck, ‘Artisans as knowledge workers: craft and creativity in a long-term perspective’, *Geoforum*, **99**, 227–237 (2019), at pp. 230 and 234.

103 Jacob, *op. cit.* (note 26), ff. 328r–329v.

104 Elaine Leong, ‘Collecting knowledge for the family’, *Centaurus* **55**, 81–103 (2013), at p. 91.

105 Herbert, *op. cit.* (note 4), p. 104.

106 Schaffer and Shapin, *op. cit.* (note 97), p. 59.

107 Kowalchuk, *op. cit.* (note 5), p. 34.

108 Werrett, *op. cit.* (note 36), p. 104.

109 Leong and Rankin refer to the ‘near-synonymous status of the words [“experiment” and “experience”] before the seventeenth century’. Leong and Rankin, *op. cit.* (note 4), p. 168.

110 Jacob, *op. cit.* (note 26), f. 335r.

111 *Ibid*.

112 Hyde, *op. cit.* (note 30), f. 31v.

113 Schaffer and Shapin, *op. cit.* (note 97), p. 64. See also Eamon, *op. cit.* (note 98); Seth LeJacq, ‘The bounds of domestic healing: medical recipes, storytelling and surgery in early modern England’, *Soc. Hist. Med.* **26**(3), 451–468 (2013).

114 Perrot, *op. cit.* (note 68), f. 34r.

115 LeJacq, *op. cit.* (note 113), p. 453.

116 Francisco Alonso-Almeida, ‘Genre conventions in English recipes, 1600–1800’, in DiMeo and Pennell, *op. cit.* (note 43), pp. 68–90, at pp. 79–80.

117 Blair, *op. cit.* (note 27), p. 64.

118 Hyde, *op. cit.* (note 30), f. 27v.

119 Carr, *op. cit.* (note 32), f. 23r.

120 Chantrell, *op. cit.* (note 53), f. 66v.

121 Leong and Pennell, *op. cit.* (note 15), p. 134.

122 Hyde, *op. cit.* (note 30), f. 36r.

123 LeJacq, *op. cit.* (note 113), p. 456.

124 Steven Shapin, ‘The house of experiment in seventeenth-century England’, *Isis* **79**(3), 373–404 (1988), at p. 375.

125 Schaffer and Shapin, *op. cit.* (note 97), pp. 55–57.

126 Simon Schaffer, ‘Natural philosophy and public spectacle in the eighteenth century’, *Hist. Sci.* **21**, 1–43, (1983), at p. 1.

127 Godfrey, *op. cit.* (note 30), f. 122v.

128 *Ibid*., f. 113r.

129 Hyde, *op. cit.* (note 30), ff. 47r and 69r.

130 Deborah Harkness, ‘Managing an experimental household: the Dees of Mortlake and the practice of natural philosophy’, *Isis* **88**(2), 247–262 (1997), at p. 262.

## Appendix: transcribed preserved walnut recipes^131^

**Bridget Hyde's recipe book, MS.2990, f. 48v**

To Pickle Walnuts

Take green Walnuts, pick y^m^ from the

stalks, put y^m^ in cold water, & set y^m^ on a

great fire till y^e^ outward skin begin to peel

off, then wipe them of w^th^ a close corse [above] cloth; y^n^ put

y^m^ into a Tub & put salt & water to them,

changing them ten or 12 dayes till the bitterness

or discolour be gone.

  The Pickle           Take halfe

white wine & halfe the best wine vinegar

according to y^e^ quantity you do of nuts; set it

on the fire; Boil it halfe an hour, a little

before you take it off put in white pepper,

ginger, Cloves & Mace of each a like

quantity enough to make it strong of the

spice, & a few Bay leaves; then have your

nutts wiped dry & put them into y^e^ Pickle —

whilst it is boiling & imediately take them of

the fire & set y^m^ by & cover it close, & w^n^ tis cold put

y^m^ into a new earthen pot well glazed tying y^m^ close

over w^th^ a peice of leather

**Elizabeth Godfrey's recipe book, MS.2535, unpaginated**

To pickle greane walnutts

Take green walnutts before they have meate in them

boyle them in water and salt as they couller the water

shift them this doe 2 or 3 times scuming [?] them cleane as

it risheth [?] boyle them till they are a little softish but

not to be to soft take them up and wipe them take

them when they are cold and put them in a stone pott

laying as much dill as will cover the bottom of the

bottom of the pott then put in as many wallnutts

as will cover the dill on the nutts stew a handful of salt

in this manner fill the pott 3 quarters full putt in some cloves

mace and a little hole peper and some cloves of garlick

or for want of garlick some shurlotte but garlick is best

lay dill att top and some salt then fill the with y^e^ best

white wine vineger tie it up close from the ayre and

let itt stand 2 or 3 months before you open itt if the

pickle be decayed putt in fresh vinegar poureing

the old away putt in fresh spice and garlick likewise

you may scrape of the skin when you eat them if they

be to keep all the yeare you must not boyle them onely

scald them or shift them in cold water every day for

a month together till the bitternes be out of them this

way usually pracktis [?] the water must be fresh shifted

every day. June is the time to pickle them.

**Elizabeth Jacob's recipe book, MS 3009, f. 273r**

To Pickle Walnuts

Take your walnuts, a week before midsommer before the shells be hardend

and put them in water, for 9 days together, shifting them twice A day

then give them A boile in Springwater, and when They are boiled put

them A dreining, then Take The best whit wine vinegar, and put to it some

hole peper, some Cloves and mace, and some sherlots or garlick, and

give all this A boile together, and put your walnuts in an Earthen pot

and put your liquor hott upon them, and when they are cold, cover them

very close, and be sure you keep them in A coole place, and if at any

time you shall see the liquor below the walnuts, you must poure the

liquor from them, and boile it again, with some fresh vinegar, and poure

it hott as before. you must be sure to pull the walnuts, for if they be

bruised they will not keep/

**Bridget Parker's recipe book, MS.3768, f. 30v**

To preserve walnuts physichaly

Take of ye largest walnuts after midsomer [this line crossed out]

& run a [?oodk/boek] throw ym both ways y^n^ put y^m^ [this line crossed out]

into water to keep ye colour as you doe y^m^ y^n^ [this line crossed out]

Take walnuts about midsumer & cut of

The ends y^n^ boyle y^m^ in 2 waters but the

biternes must not be out to much nor they to

tender y^n^ to every pound of nutts put a pound

of Brown suger & half a nine pint of water

boyle y^e^ suger & scume it cleane & when it is

almost cold put in y^e^ nuts wip y^m^ dry & cut

y^m^ in y^e^ sids w^th^ a pen Knife to y^e^ midle &

stick half a clove in each of y^m^ y^n^ boyle y^m^

a litle & boyle the sirrop to a thicknes & poure

over y^m^ when att any time it grows thine boyle

it againe & poure on y^m^

Take 2 att night when you are [?costine] or be

for the taking of a physick it is holsom against

infection consumption or weakness Z

**Mary Miller's recipe book, MS.3547 f. 39r**

To preserve Walnutes green

Take green walnuts & steep y^m^ all night in

Faire watter in y^e^ morning pare y^m^ and boile y^m^

In a skillet of faire watter for a [little] while

Y^n^ shift y^m^ into another skillet of water, for

[feard] they should grow balcke & soe shifting

y^m^ let y^m^ boyle till they be tender through

to [thake] a clove at both ends, drying every

one of ym w^th^ a cleane cloth as yow [sticke] your

cloves & so put y^m^ into the sirrop take a

pound & quarter of sugar to every pound

of walnuts, & to every pound of sugar take

a pint of rose water & a graine either

of ambergrease or muske, & bruise it very

well in y^e^ rosewater & y^n^ put it in your

preserved walnuts, & let y^m^ stand till

they be cold, y^n^ pott y^m^ & so yow may keep

y^m^ a yeare & or fare /

